# *Plastrum Testudinis* Extract Promotes Endogenous Bone Marrow Mesenchymal Stem Cell Migration in Osteoporotic Fracture Repair Partly by Activating the SDF‐1/CXCR4 Axis

**DOI:** 10.1155/sci/3033093

**Published:** 2026-05-19

**Authors:** Xingda Chen, Hang Zhuo, Peng Zhang, Jizhi Ma, Guibo Liang, Riwei Tan, Yan Gong, Yifei Wang, Weicheng Qin, Jiahui Dai, Zilan Zhong, Hongze Wang, Ruicheng Lin, Zheng Kuang, Liang De, Yongchao Tang, Jinyong Ding, Zixian Wu, Xiang Yu

**Affiliations:** ^1^ Guizhou University of Traditional Chinese Medicine, Guiyang, 550000, China, gzu.edu.cn; ^2^ Department of Orthopaedics, Suzhou TCM Hospital Affiliated to Nanjing University of Chinese Medicine, Suzhou, 215009, China, njucm.edu.cn; ^3^ The Second Clinical Medical College, Guangzhou University of Chinese Medicine, Guangzhou, 510006, China, gzucm.edu.cn; ^4^ Department of Spine, Guangzhou University of Traditional Chinese Medicine First Affiliated Hospital, Guangzhou, 510120, China; ^5^ Department of Spine, Guangdong Clinical Research Academy of Chinese Medicine, Guangzhou, 510120, China; ^6^ Department of Orthopaedics, Lingnan Medical Research Center, Guangzhou, 510120, China; ^7^ Department of Orthopaedics, Beijing University of Chinese Medicine, Beijing, 100000, China, bucm.edu.cn; ^8^ Acupuncture and Rehabilitation Clinical Medicine Institute, Guangzhou University of Chinese Medicine, Guangzhou, 510120, China, gzucm.edu.cn; ^9^ Department of Metabolism and Endocrinology, The Second Xiangya Hospital of Central South University, Changsha, 410011, China, csu.edu.cn; ^10^ Department of Acupuncture, The Second Affiliated Hospital of Guangzhou University of Chinese Medicine, Guangzhou, 510405, China, gzucm.edu.cn; ^11^ Shenzhen Clinical Medical College, Guangzhou University of Chinese Medicine, Guangzhou, 510405, China, gzucm.edu.cn

**Keywords:** bone marrow mesenchymal stem cells, osteoporotic bone repair, *Plastrum Testudinis* extract, SDF-1/CXCR4 axis, stem cell migration, traditional Chinese medicine

## Abstract

Osteoporotic fracture (OPF) is a major global health concern, particularly among aging populations, and the compromised migration and osteoblast differentiation potential of bone marrow mesenchymal stem cells (BMSCs) in osteoporosis significantly hinder fracture repair. The stromal cell‐derived factor‐1 (SDF‐1)/C‐X‐C chemokine receptor 4 (CXCR4) axis is vital for guiding BMSC migration and bone regeneration. However, *Plastrum Testudinis* (PT) extract (PTE), a traditional Chinese medicine (TCM) derived from turtle shell, has revealed promise in promoting BMSC proliferation and differentiation, although its mechanism of regulating endogenous stem cell migration in OPF remains unclear. To address this, we combined bioinformatics screening, molecular docking, clinical tissue analysis, and in vivo experiments to identify key pathways via Gene Ontology (GO) and Kyoto Encyclopedia of Genes and Genomes (KEGG) analyses. We established a bilateral ovariectomy‐induced osteoporotic mouse model with tibial defects and treated the mice with PTE alone or with the CXCR4 inhibitor AMD3100, followed by histological, micro‐CT, immunofluorescence, and in vitro Transwell assays. Bioinformatics analysis revealed enriched chemokine signaling pathways and identified CXCR4 as a key target of PTE. Molecular docking confirmed stable binding between PTE’s active components (phenylalanine, methionine, threonine, and aspartic acid) and CXCR4. PTE significantly improved bone volume/total volume (BV/TV) and trabecular microarchitecture in osteoporotic tibial defect (OTD) mice, reversed reduced SDF‐1/CXCR4 expression in osteoporotic tissues, and enhanced BMSC migration and SDF‐1/CXCR4 mRNA levels in vitro; these effects were blocked by AMD3100. In summary, PTE effectively rescues the migration deficit of endogenous BMSCs under osteoporotic conditions by targeting the SDF‐1/CXCR4 axis. This enhanced recruitment accelerates OPF healing, highlighting the potential of PTE as a regenerative therapeutic strategy.

## 1. Introduction

Osteoporotic fracture (OPF) and the associated bone defects often exhibit delayed or impaired healing due to persistent oxidative stress and immune dysregulation in the local microenvironment. They represent a major cause of disability and mortality among elderly patients with fractures, imposing a substantial burden on families and society. It is projected that by 2050, the number of newly diagnosed OPF cases in China will reach 5.99 million, with the corresponding medical expenditure estimated to increase to 174.5 billion RMB [1]. Among these conditions, osteoporotic bone defects constitute a particularly challenging clinical problem in managing OPF, for which effective therapeutic strategies remain limited [2]. Fracture healing critically depends on the migratory capacity and osteogenic differentiation potential of bone marrow mesenchymal stem cells (BMSCs) [3–5]. However, these endogenous reparative processes are markedly impaired in patients with osteoporosis, resulting in delayed or insufficient bone regeneration [6, 7]. Mechanistically, Liu et al. [8] demonstrated that BMSCs derived from ovariectomized osteoporotic rats exhibit significantly downregulated CXCR4 expression and attenuated p‐AKT signaling, leading to a marked deficit in their chemotactic migration towards SDF‐1.

Although transplantation of exogenous BMSCs has been explored as a therapeutic strategy, its clinical translation remains limited due to immune rejection and difficulties in controlling lineage commitment [9–11]. Consequently, strategies aimed at activating endogenous BMSCs recruitment have gained increasing attention. Furthermore, alongside cell‐based and molecular strategies, developing advanced bone defect repair materials, particularly 3D‐printed bioceramic scaffolds and biomimetic hierarchical structures, has exhibited considerable translational potential for the reconstruction of complex bone defects [12–15]. The SDF‐1/CXCR4 axis is vital for guiding BMSC chemotaxis and promoting bone repair [16–18].

**Figure 1:**
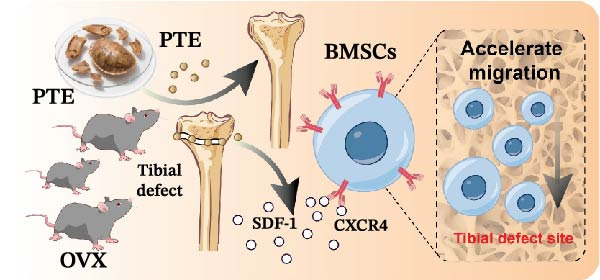
Schematic illustration of BMSC activation, proliferation, and osteoblast differentiation in response to tibial defect.

Recent studies further suggest that traditional Chinese medicine (TCM) can modulate this pathway to enhance tissue regeneration [19]. Previous studies have demonstrated that TCM has significant advantages in OPF treatment [20, 21]. *Plastrum Testudinis* (PT), a traditional animal‐derived medicinal ingredient, is commonly used for *bushengqianggu* (tonifying the kidneys and strengthening the bones). Its water extract (PT extract [PTE]) has demonstrated osteogenic and bone‐strengthening effects in several experimental studies [22, 23]. In vitro evidence reveals that PTE enhances BMSC proliferation and osteogenic differentiation [7, 24, 25]. However, whether PTE promotes endogenous BMSC migration through the SDF‐1/CXCR4 axis has not been systematically investigated. This study used a multitiered strategy, bioinformatic prediction, clinical tissue validation, an osteoporotic tibial defect (OTD) animal model, and mechanistic cellular assays to elucidate how PTE activates SDF‐1/CXCR4 axis and accelerates OTD healing by enhancing BMSC recruitment. The overall hypothesis is illustrated in Figure 1.

In an ovariectomized mouse model of tibial defect, PTE treatment enhances BMSC migration to the defect site. This effect is mediated by upregulating the SDF‐1/CXCR4 axis, which drives BMSC recruitment to the injured area to facilitate repair.

## 2. Materials and Methods

### 2.1. Patients

This study was approved by the Ethics Committee of the First Affiliated Hospital of Guangzhou University of Chinese Medicine (Approval Number JY2024‐200). Patients who underwent lumbar interbody fusion surgery at the Department of Spine Surgery of our hospital between December 25, 2024 and July 1, 2025, were retrospectively enrolled. Intraoperatively discarded vertebral bone tissue specimens were collected for subsequent analysis. All participants were postmenopausal women who voluntarily participated in the study and provided their written informed consent. Eligible patients were required to have vertebral bone tissue specimens obtained during surgery and complete clinical records, including laboratory and imaging data. Patients with diseases known to affect bone metabolism, including malignancy, thyroid disorders, or autoimmune diseases, or those who received medications that may influence bone metabolism, were excluded. These medications included antiosteoporosis treatments (e.g., vitamin D, calcium carbonate, calcitonin, denosumab, or teriparatide) and other drugs affecting bone metabolism (e.g., glucocorticoids) (Supporting Information 1: Table S1).

### 2.2. Reagents

The reagents used in this study included AMD3100 (HY‐10046, MCE), anti‐CD29 (GB115629‐50, Servicebio), anti‐CD90 (GB113753‐50, Servicebio), anti‐SDF‐1 (GB11624‐50, Servicebio), anti‐RUNX2 (GB115631‐50, Servicebio), and anti‐CXCR4 (AF5279, Affinity).

### 2.3. Drug Tests and Treatment

PT was acquired from a qualified clinical institution (Batch Number: KG240301) (Supporting Information 2: Form 1). In total, 1000 g of crushed PT was boiled in 1 L of pure water for 1 h. The extraction was repeated thrice on the residue, and the collected supernatants were adjusted to a final volume of 1 L. For in vivo experiments, the intragastric dose of PT extract (PTE group) was 4 g/kg/day [26], and the subcutaneous dose of AMD3100 was 2.184 mg/kg/day, calculated based on the human‐mouse body surface area ratio. For in vitro cellular studies, PTE (20 μg/mL) and AMD3100 (5 μg/mL) concentrations were determined following previous reports, respectively [26, 27].

### 2.4. Mice

A total of 48 female C57BL/6 mice (2 months old, 18–22 g) were obtained from the Experimental Animal Center (SCXK(Y)2018‐0034). Housing, model establishment, and herbal interventions were conducted at the Laboratory Animal Center (SYXK(Y)2018‐0092). The experimental procedures were approved by the Institutional Ethics Committee (GZTCMF1‐20240110). First, mice were randomized into three groups (*n* = 6/group): Sham‐operated (SHAM), ovariectomized OTD, and PTE treatment (PTE). OTD and PTE groups underwent bilateral ovariectomy, while SHAM animals received only skin incisions. Six weeks post‐ovariectomy [28], a 1‐mm unicortical defect was drilled in the right tibia to establish a highly reproducible focal bone defect model [29]. We used a 1‐mm tibial drill‐hole defect, rather than a true fracture, to isolate early BMSC migration and intramembranous ossification, avoiding confounding mechanical instability to reveal the cellular mechanisms of PTE. Penicillin was administered for 3 days postsurgery (20,000 IU/mouse/time). The PTE group received PTE by gavage; SHAM and OTD groups received saline. Tissues were collected 2 weeks after the intervention. Thirty 2‐month‐old female C57BL/6 mice were stratified by body weight using the RAND function in Excel and randomly allocated into five groups (*n* = 6/group): SHAM, OTD, PTE, AMD (CXCR4 inhibitor AMD3100), and P + A (PTE + CXCR4 inhibitor AMD3100). AMD3100 was administered subcutaneously in AMD and P + A groups. PTE was given by gavage in PTE and P + A groups. The other groups received equal‐volume saline injections. After 2 weeks, the tissues were harvested.

### 2.5. Target Gene Prediction, Bioinformatic Analysis, and Network Construction

The active constituents of PTE, identified and confirmed via ultra‐performance liquid chromatography, were used to predict potential therapeutic targets using pharmacological databases (including TCMSP/SwissTargetPrediction) (Supporting Information 3: Figure S1). Concurrently, genes associated with “osteoporosis” and “fracture” were independently retrieved from the GeneCards database (https://www.genecards.org/). To ensure strong disease association and filter out low‐relevance noise, the retrieved targets were ranked by their relevance scores, and the top 2000 highest‐scoring targets for each condition were selected. The intersection between PTE‐predicted targets and filtered disease targets was identified as the shared therapeutic gene set, and a Venn diagram was generated using the Microbioinformatics Platform (http://www.bioinformatics.com.cn/).

To identify the relationships among the intersection targets and establish target prioritization, the STRING database (https://string-db.org/) was used to obtain protein–protein interaction (PPI) data, with the species limited to *Homo sapiens*. The PPI network was constructed using Cytoscape software (Version 3.7.2; http://www.cytoscape.org/). Network topology analysis was conducted using the NetworkAnalyzer plugin to calculate core topological parameters, including the degree of each node, thereby providing quantifiable evidence for prioritizing key hub genes, including CXCR4, within the PT‐compound‐target‐disease network.

Gene Ontology (GO) and Kyoto Encyclopedia of Genes and Genomes (KEGG) enrichment analyses of the intersection targets were performed using the clusterProfiler package in R, with a significance threshold of *p*  < 0.05. The ggplot2 package and Cytoscape software were used for visualization. GO enrichment analysis included molecular function (MF), cellular component (CC), and biological process (BP); the top 10 terms in each category were presented. The top 10 enriched KEGG pathways were also displayed.

### 2.6. Molecular Docking Analysis

Ligand structures were constructed in ChemDraw and exported in .mol2 format, followed by hydrogenation using the Open Babel software. Protein receptor structures were obtained from the Protein Data Bank, processed in PyMOL to remove water molecules and impurities, hydrogenated, and converted to .pdbqt format using AutoDockTools with defined charge and grid box settings. Docking was performed using AutoDock Vina (Version 1.2.2; http://autodock.scripps.edu/), and binding modes and affinities (kcal/mol) were analyzed in PyMOL software.

### 2.7. Micro‐CT Analysis

Micro‐CT scanning was performed at 80 kV, 100 μA, and 10‐μm slice thickness. Reconstruction (NRecon; Version 1.7) and quantitative analysis (CTAn; Version 1.1.6) were performed for the region of interest, assessing bone volume/total volume (BV/TV), Tb.Th, Tb.N, and Tb.Sp.

### 2.8. Histological Processing and Staining

Right tibias were collected after euthanasia, rinsed in phosphate‐buffered saline, fixed in 4% paraformaldehyde for 48 h, washed, and transferred to 70% ethanol. Samples were decalcified in 10% EDTA for 3–5 weeks, dehydrated, cleared, embedded, and sectioned (4 μm).

Hematoxylin and eosin (H&E) staining was performed for morphological evaluation. Tartrate‐resistant acid phosphatase (TRAP) staining was used to identify osteoclast activity. Immunofluorescence and immunohistochemistry were used to detect functional protein expression following the kit instructions.

### 2.9. Histological Analysis of Clinical Specimens

Human bone tissues were fixed in 4% paraformaldehyde for 48 h, decalcified with EDTA, dehydrated, and processed for H&E, TRAP, and immunofluorescence staining using the same procedures described for mouse samples.

### 2.10. Transwell Migration Assay

BMSC migration was assessed using Transwell chambers (24‐well, 8‐μm pores; Corning CLS3464). BMSCs (1 × 10^4^ cells) were seeded into the upper chamber with α‐MEM containing 1% FBS, while the lower chamber contained α‐MEM with 10% FBS. PTE was applied at 20 μg/mL to BMSCs. Four groups were established: CON (no PTE), PTE (20 μg/mL PTE), ADM (ADM3100 [5 μg/mL] ADM + PTE (ADM3100 added first, followed 12 h later by PTE) [27]. After 24 h, the migrated cells were stained with crystal violet and quantified. SDF‐1 and CXCR4 mRNA levels were measured by RT‐qPCR (Supporting Information 4: Table S2).

### 2.11. Statistical Analysis

Data were analyzed using the Statistical Package for the Social Sciences software (Version 25.0), and graphs were generated using GraphPad Prism software (Version 8.3.0). Continuous variables are expressed as mean ± standard deviation. Statistical significance for comparisons between two independent groups was determined using an unpaired, two‐tailed Student’s *t*‐test. For comparisons among three or more groups, a one‐way analysis of variance was performed, followed by Tukey’s post hoc test. A *p*  < 0.05 was considered statistically significant.

## 3. Results

### 3.1. Bioinformatic Prediction of the Mechanisms by Which PTE May Promote Osteoporotic Bone Repair

To identify the key molecular targets involved in OPF and to explore whether the active constituents of PT interact with the chemotactic factor CXCR4, we first performed a series of bioinformatic analyses. Genes associated with “osteoporosis” and “fracture” were retrieved separately from the GeneCards database, and the top 2000 targets for each term were intersected, yielding 1040 shared candidate genes.

**Figure 2:**
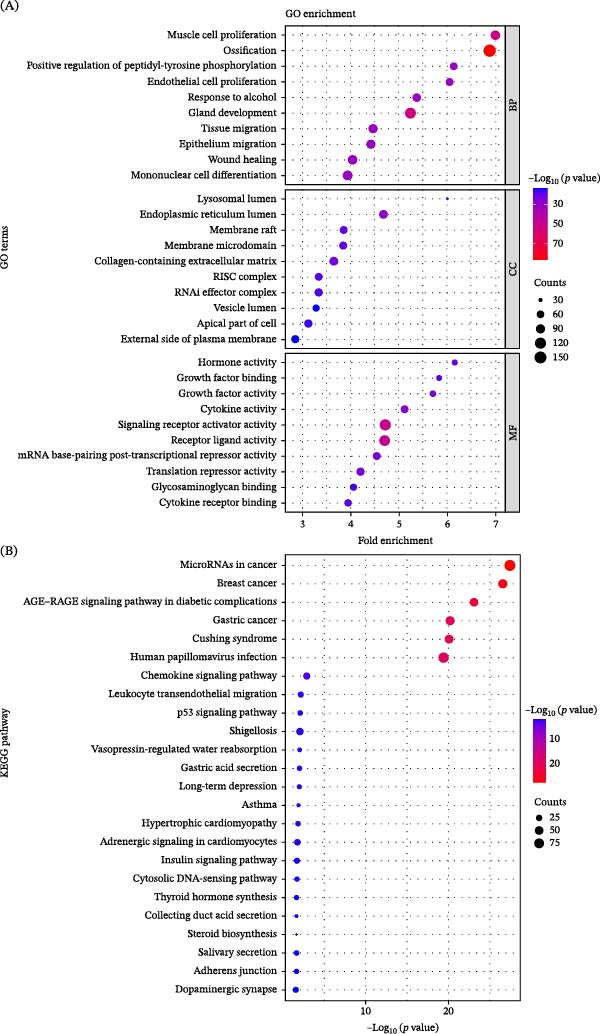
Gene functional annotation analysis. (A) Gene Ontology (GO) enrichment analysis. (B) Kyoto Encyclopedia of Genes and Genomes (KEGG) pathway enrichment analysis.

GO enrichment analysis revealed distinct functional signatures across BP, CC, and MF categories. The top 10 terms within each category (30 entries in total) mainly highlighted processes relevant to musculoskeletal development and repair. BP terms were enriched in muscle cell proliferation, ossification, positive regulation of peptidyl‐tyrosine phosphorylation, endothelial cell proliferation, tissue and epithelial migration, wound healing, and mononuclear cell differentiation. CC mapping localized targets predominantly to the lysosomal lumen, endoplasmic reticulum lumen, membrane rafts, collagen‐containing extracellular matrix, RNA‐induced silencing complex, and vesicle lumen. MF enrichment analysis emphasized hormone activity, growth factor binding/activity, cytokine activity, signaling receptor activation, mRNA posttranscriptional repression, and glycosaminoglycan/cytokine receptor binding (Figure 2A).

KEGG pathway analysis revealed enrichment in signaling cascades associated with tissue regeneration and inflammation, including microRNAs in cancer, chemokine signaling, leukocyte transendothelial migration, p53 signaling, the AGE‐RAGE pathway, and several cancer‐related pathways (Figure 2B).

**Figure 3:**
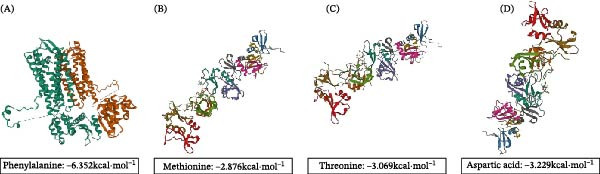
Molecular interaction analysis. (A–D) Molecular docking analysis between different PTE active ingredient amino acids (phenylalanine (A), methionine (B), threonine (C), aspartic acid (D)), and target proteins.

To further predict the relationship between PTE constituents and CXCR4, molecular docking was conducted. Four representative amino acid‐type components in PTE, phenylalanine, methionine, threonine, and aspartic acid, displayed favorable binding affinities with CXCR4 and formed stable interaction modes within the predicted binding pocket (Figure 3A–D) [30].

### 3.2. In Vivo Evidence That PTE Promoted OTD Repair Through Enhanced Endogenous BMSC Migration

To validate the bioinformatic predictions, we assessed the reparative effects of PT in an ovariectomy‐induced osteoporotic mouse model. A standardized tibial defect was created 6 weeks after ovariectomy to mimic OTD, while SHAM animals underwent skin incision only. After 2 weeks, micro‐CT imaging revealed abundant new bone formation in the defect area in SHAM mice, whereas OTD mice exhibited persistent cavitation with sparse and thin trabeculae. Quantitative analysis confirmed significant reductions in BV/TV and Tb.N, together with an elevation in Tb.Sp, in OTD mice versus SHAM (*p* < 0.05), verifying successful model induction (Supporting Information 5: Figure S2).

**Figure 4:**
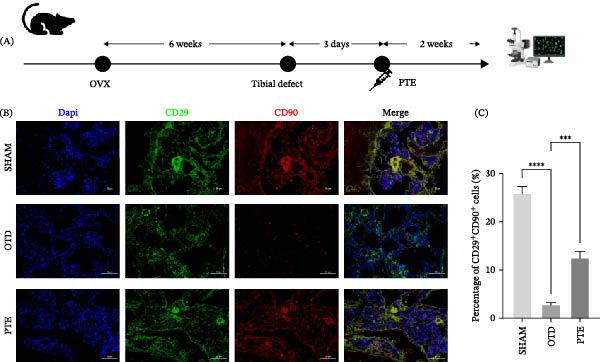
Establishment of the mouse tibial bone defect model and analysis of bone marrow mesenchymal stem cells surface marker expression (CD29, CD90). (A) Schematic diagram of the experimental timeline. (B) Representative immunofluorescence staining images showing DAPI (blue, nuclei), CD29 (green), and CD90 (red). Merge indicates the overlay. Scale bars, 250 µm. (C) Quantitative analysis of the percentage of CD29^+^CD90^+^ cells (%) in each group (*n* = 6). Statistical significance was determined by one‐way ANOVA followed by Tukey’s post hoc test.  ^∗∗∗∗^ *p* < 0.0001 vs. SHAM group,  ^∗∗∗^ *p* < 0.001 vs. OTD group.

PTE treatment markedly improved defect healing. The defect cavity decreased in size, and micro‐CT metrics demonstrated increased BV/TV (*p* < 0.05), elevated Tb.N (*p* < 0.05), and reduced Tb.Sp (*p* < 0.05) compared with OTD (Supporting Information 5: Figure S2). Correspondingly, immunofluorescence revealed that expression levels of BMSC markers CD29 and CD90 were diminished in OTD lesions but restored after PTE administration (Figure 4A–C).

### 3.3. Clinical Bone Specimens Revealed Reduced SDF‐1/CXCR4 Expression in Patients With Osteoporosis

**Figure 5:**
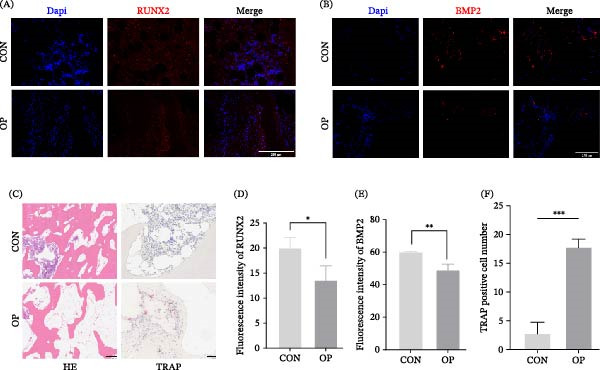
Osteogenic and osteoclastic activity in bone tissue from nonosteoporotic and osteoporotic patients. (A, B) Representative immunofluorescence images showing RUNX2 (A) and BMP2 (B) expression in bone tissue. Scale bars, 250 µm. (C) Representative images of H&E staining (scale bars, 500 µm) and TRAP staining (scale bars, 100 µm) of bone tissue. Arrows indicate osteoclasts. (D, E) Quantitative analysis of RUNX2 (D) and BMP2 (E) fluorescence intensity in each group (*n* = 3). (F) Quantification of TRAP‐positive cells in each group (*n* = 3). Statistical significance was determined by unpaired two‐tailed Student’s *t*‐test.  ^∗^ *p*  < 0.05,  ^∗∗^ *p*  < 0.01,  ^∗∗∗^ *p*  < 0.001 vs. nonosteoporotic group.

**Figure 6:**
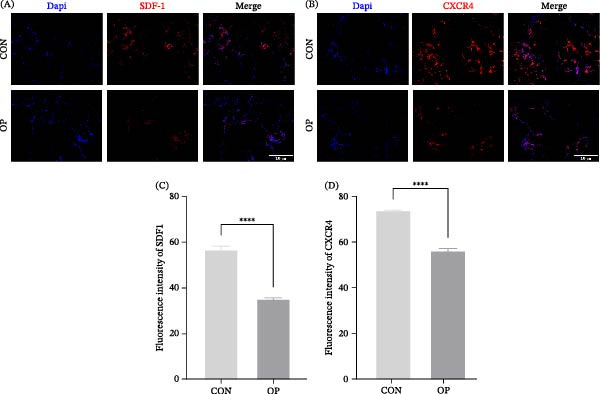
Expression of SDF‐1 and CXCR4 in bone tissue from nonosteoporotic and osteoporotic patients. (A) Representative images of SDF‐1 immunofluorescence staining. Scale bars, 250 µm. (B) Representative images of CXCR4 immunofluorescence staining. Scale bars, 250 µm. (C, D) Quantitative analysis of SDF‐1 (C) and CXCR4 (D) fluorescence intensity in each group (*n* = 3). Statistical significance was determined by an unpaired two‐tailed Student’s *t*‐test.  ^∗∗∗∗^ *p* < 0.0001 vs. nonosteoporotic group.

To assess the clinical relevance of SDF‐1/CXCR4 signaling, bone samples from patients with OP and CON were examined. Immunofluorescence revealed reduced expression of the osteogenic markers RUNX2 and BMP2 in OP samples (Figure 5A,B,D,E). Histology confirmed characteristic osteoporotic architecture, with disrupted and thinned trabeculae compared with the dense and organized structure in CON tissues. TRAP staining revealed increased osteoclast numbers and activity in OP specimens (Figure 5C,F). Notably, SDF‐1 and CXCR4 were significantly downregulated in OP bone, suggesting compromised stem‐cell chemotaxis and osteogenic potential (Figure 6A–D).

### 3.4. PTE Restored SDF‐1/CXCR4 Signaling and Enhanced BMSC Migration to Accelerate Bone Repair In Vivo

To determine whether the effects of PTE rely on SDF‐1/CXCR4 signaling, additional groups receiving CXCR4 antagonist AMD3100 (AMD group) or combined AMD3100 and PTE (P + A group) were established (Figure 7A). Micro‐CT and histological evaluation demonstrated that AMD3100 significantly impaired bone repair, with BV/TV reduced to 5.116% ± 2.31% and severe deterioration of trabecular microarchitecture. Conversely, P + A mice exhibited substantial improvement, with BV/TV reac–hing 17.184% ± 1.246%, and significant increases in Tb.Th and Tb.N (*p* < 0.05) relative to AMD (Figure 7B,D–G).

Figure 7PTE upregulates SDF‐1/CXCR4 expression in the mouse tibial bone defect region. (A) Schematic diagram of the experimental timeline. (B) Three‐dimensional micro‐CT reconstruction images of the tibial defect region. (C) Representative immunofluorescence staining images showing DAPI (blue, nuclei), SDF‐1 (green), and CXCR4 (red). Merge indicates the overlay. Scale bars, 200 µm. (D) Quantitative analysis of the percentage of CD29^+^CD90^+^ cells (%) in each group. (E–H) Quantitative micro‐CT analysis of tibial defects in mice: bone volume/total volume (BV/TV, E), trabecular number (Tb.N, F), trabecular thickness (Tb.Th, G), and trabecular separation (Tb.Sp, H) (*n* = 6). Statistical significance was determined by one‐way ANOVA followed by Tukey’s post hoc test.  ^∗^ *p* < 0.05,  ^∗∗^ *p* < 0.01,  ^∗∗∗^ *p* < 0.001 compared with OTD group. ^#^ *p* < 0.05, ^##^ *p* < 0.01, ^###^ *p* < 0.001 for AMD group vs. P + A group.

**Figure:**
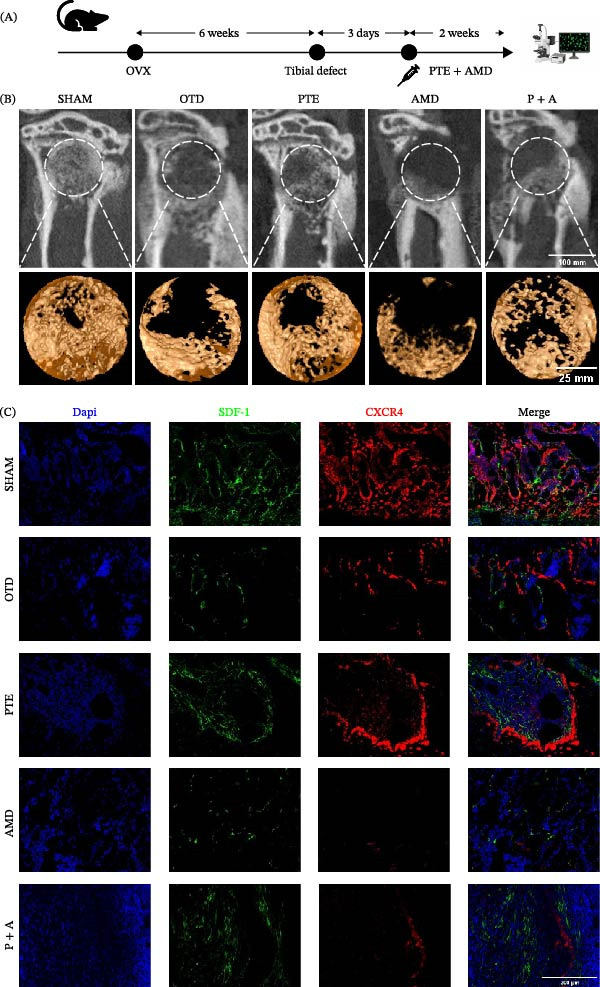

**Figure:**
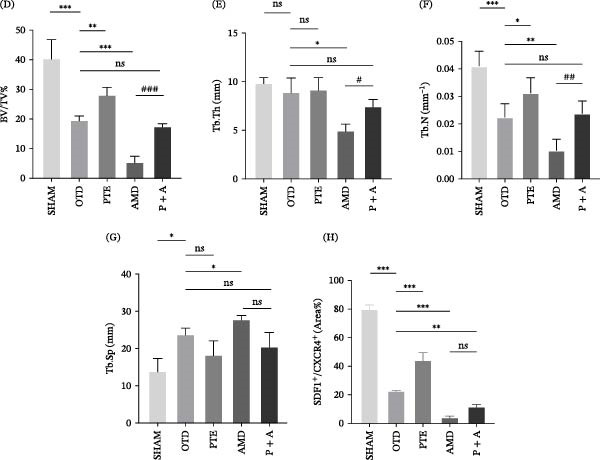

**Figure 8:**
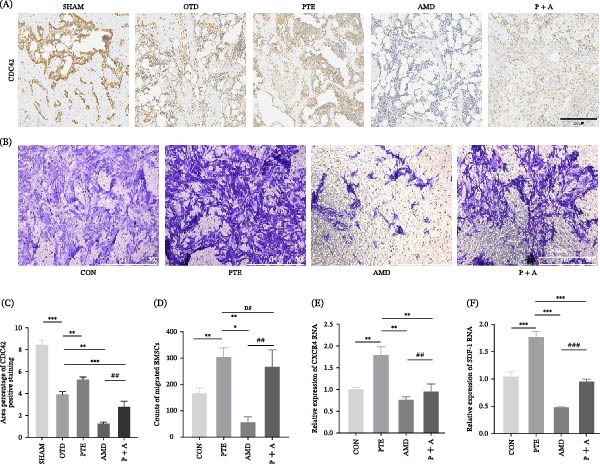
PTE promotes BMSC migration and upregulates SDF‐1/CXCR4 expression. (A) Representative immunohistochemical images of CDC42 expression in the tibial defect region. Scale bars, 200 µm. (B) Representative microscopic images of migrated BMSCs. Scale bars, 50 µm. (C) Quantitative analysis of CDC42 expression shown in the tibial defect region. (*n* = 6). (D) Quantification of migrated BMSCs (*n* = 3). (E, F) Real‐time qPCR analysis of SDF‐1 (E) and CXCR4 (F) mRNA expression levels (*n* = 3). Statistical significance was determined by one‐way ANOVA followed by Tukey’s post hoc test or an unpaired two‐tailed Student’s *t*‐test.  ^∗^ *p* < 0.05,  ^∗∗^ *p* < 0.01,  ^∗∗∗^ *p* < 0.001 compared with OTD group. ^#^ *p* < 0.05, ^##^ *p* < 0.01, ^###^ *p* < 0.001 for AMD group vs. P + A group.

Immunohistochemistry and immunofluorescence further revealed reduced expression levels of CDC42 and SDF‐1/CXCR4 in OTD and AMD groups, whereas PTE treatment restored their expression. Notably, SDF‐1 levels were markedly elevated in the P + A group (Figures 7C,H and 8A,C).

Histological assessments revealed that OTD mice formed less new bone than SHAM animals, while PTE enhanced bone formation within the defect. AMD3100 markedly suppressed osteogenesis and delayed repair, whereas concurrent PTE treatment partially reversed this suppression (Supporting Information 6: Figure S3A,C,E). TRAP staining revealed the fewest osteoclasts in the SHAM group, followed by PTE, OTD, and P + A, with AMD exhibiting the highest osteoclast burden (Supporting Information 6: Figure S3B,D).

### 3.5. In Vitro Validation That PTE Enhanced BMSC Migration via SDF‐1/CXCR4 Axis Activation

In Transwell assays, PTE significantly increased BMSC migration, reflected by dense crystal violet staining, compared with controls. AMD3100 markedly reduced cell migration, whereas P + A partially restored it. Quantitatively, PTE enhanced BMSC migration relative to CON (*p*  < 0.001), AMD reduced migration relative to PTE (*p* < 0.01), and P + A again increased migration compared with AMD (*p*  < 0.001) (Figure 8B,D). RT‐qPCR further revealed that PTE upregulated CXCR4 (*p* < 0.01) and SDF‐1 (p < 0.001) mRNA expression. AMD3100 significantly suppressed both transcripts (*p* < 0.01; *p*  < 0.0001), while P + A restored their levels (Figure 8E,F).

## 4. Discussion

Recently, accumulating evidence has demonstrated a close and functionally significant association between the migratory capacity of BMSCs and fracture healing [31]. BMSCs play an indispensable role in orchestrating the repair of osteoporotic bone defects and localized fractures, primarily by homing to injury sites and participating in subsequent bone regeneration processes [32]. Among the molecular mechanisms governing this process, SDF‐1/CXCR4 axis has been identified as a central regulator of BMSC migration [33]. Collectively, these findings provide a strong mechanistic rationale and translational framework for therapeutic strategies aimed at enhancing endogenous BMSC mobilization to improve osteoporotic bone defects and localized fracture healing outcomes.

To establish the clinical relevance of our study into impaired bone regeneration, we first evaluated osteogenic and chemotactic markers in human bone samples. Analysis of clinical bone specimens from patients with and without osteoporosis revealed a marked suppression of the SDF‐1/CXCR4 axis in osteoporotic tissue, suggesting that impaired stem‐cell migration is a critical mechanism underlying delayed union or nonunion in OPFs and osteoporotic bone defects. Having identified SDF‐1/CXCR4 axis deficit in patients, we sought a therapeutic intervention targeting this specific pathway. Consequently, we used bioinformatic analyses and a postmenopausal OTD model to explore how SDF‐1/CXCR4 axis regulates BMSC migration, enhances osteogenic differentiation, and promotes bone regeneration under osteoporotic conditions. Building upon our preliminary data and molecular docking analysis, we identified that PTE robustly upregulates SDF‐1/CXCR4 axis pathway, thereby enhancing BMSC migratory capacity and osteogenic commitment, and ultimately accelerating bone repair under osteoporotic conditions.

In OPF, the reduced migratory and osteogenic potential of BMSCs is widely recognized as a major contributor to impaired bone repair [34, 35]. Our findings indicate that the osteoporotic microenvironment is characterized by structural fragility, compromised stem cell niches, and weakened chemotactic cues, consistent with recent reports on altered endogenous stem cell dynamics during bone regeneration [36]. Direct BMSC transplantation has been proposed to support bone remodeling in patients with OPF [37]. However, the limitations of exogenous BMSC engraftment are well documented and remain a major barrier to effective clinical translation [10]. Accordingly, targeting endogenous BMSC migration represents a rational and innovative therapeutic strategy. In osteoporotic models, SDF‐1 overexpression markedly enhances BMSC homing and osteogenesis [33], whereas mutations in CXCR4, as observed in WHIM syndrome, are associated with pronounced osteoporosis [38]. Moreover, SDF‐1/CXCR4 axis has been consistently validated as a central pathway governing stem cell homing and local tissue repair in transplantation studies [37, 39]. Our analysis of patient bone samples further confirmed the significant reduction of SDF‐1/CXCR4 expression in osteoporotic tissue compared to nonosteoporotic controls.

Our research team has maintained a long‐standing focus on osteoporosis and bone regeneration [40]. In TCM, *Carapax et* PT (turtle shell, the source of PTE) is believed to “reinforce the kidney” and “strengthen bone,” aligning with its traditional indications for bone weakness. Modern studies similarly demonstrate its pro‐osteogenic activity [22, 23]. In vitro, PTE enhances BMSC proliferation and osteogenic differentiation and contributes to accelerated fracture healing [7, 24, 25]. Molecular docking further predicted a strong affinity between key PTE components (phenylalanine, methionine, threonine, and aspartic acid) and CXCR4, leading us to hypothesize that PTE may exert its therapeutic effects on OTD by activating SDF‐1/CXCR4 axis.

Previous reports also suggest that PTE regulates stem‐cell migration and osteogenic signaling pathways [41–43]. To explicitly connect our mechanistic predictions to functional restorative outcomes, we evaluated PTE treatment in both animal models and cellular assays. Our in vivo experiments demonstrated that PTE significantly improved bone defect healing in osteoporotic rats. Furthermore, our in vitro migration assays confirmed that PTE directly promotes endogenous BMSC recruitment marked by CD29/CD90 [44]. Notably, blockade of CXCR4 with AMD3100 abrogated these effects, confirming that SDF‐1/CXCR4 axis is essential for PTE activity. Nonetheless, bone regeneration is a highly complex process that involves angiogenesis, immunomodulation, and coupled osteoblast‐osteoclast dynamics. Prior studies have revealed that SDF‐1/CXCR4 axis may also modulate endothelial cell behavior, mobilize stem‐cell‐derived exosomes, and regulate chemotactic signaling [33, 45]. Therefore, PTE may influence angiogenesis, immune modulation, and osteoblast‐osteoclast coupling. We emphasize that while our data demonstrates a central role for BMSC migration, other mechanisms may synergistically contribute to the overall bone‐regenerative effect of PTE.

Collectively, our findings demonstrate that PTE activates SDF‐1/CXCR4 axis to enhance BMSC migration and osteogenesis, thereby accelerating bone defect repair under osteoporotic conditions. This study provides a mechanistic rationale associating traditional herbal therapeutics with endogenous stem‐cell‐mediated bone regeneration and offers theoretical support for future integration of herbal bioactive compounds with bone‐repair biomaterials, including graft substitutes, implants, and scaffolds.

## 5. Limitations

Despite its multidimensional design, this study has several limitations. First, although molecular docking predicted several potential active constituents of PTE (e.g., phenylalanine, methionine, threonine, and aspartic acid), experimental confirmation through LC‐MS/MS identification, fractionation, and dose‐response analysis remains necessary. Second, although decreased SDF‐1/CXCR4 expression was confirmed in clinical samples, large‐scale randomized controlled trials are still needed to evaluate the therapeutic efficacy of PTE in patients with OPF. Third, although a tibial 1‐mm defect model was established, it does not fully replicate the complexity of human OPF, including weight‐bearing, internal fixation, or abnormal microenvironmental cues. Future studies should consider clinically relevant fracture models, including load‐bearing bone fractures, comminuted fractures, or biomaterial‐assisted repair models. Additionally, using lineage‐tracing mouse models or single‐cell RNA sequencing would provide more definitive evidence of the specific cell populations involved.

## 6. Conclusion

Through bioinformatic analysis, molecular docking, animal modeling, BMSC migration assays, and human bone‐tissue evaluation, this study systematically demonstrates that PTE enhances BMSC migration and osteogenic differentiation by activating SDF‐1/CXCR4 axis, thereby accelerating tibial‐defect healing under osteoporotic conditions. These findings highlight the essential role of stem cell homing and microenvironmental optimization in OTD repair and position PTE as a promising herbal therapy in modern bone regeneration strategies. SDF‐1/CXCR4 axis represents a novel and clinically meaningful paradigm for treating OPFs using PTE.

## Funding

This work was supported by the projects of the National Natural Science Foundation of China (Grant 82305264), Basic and Applied Basic Research Topics (Young Doctoral “Start” Project) (Grant 2024A04J4331), Scientific Research Project of Traditional Chinese Medicine Bureau of Guangdong Province (Grant 20251107), and Young, Middle‐aged Key Talent Training Project of the First Affiliated Hospital of Guangzhou University of Chinese Medicine‐Young Talents (Grant 2023QY13), and 2024 Doctoral Student Innovation Capacity Enhancement Project of “Open Bidding for Selecting the Best Candidates” from Guangzhou University of Chinese Medicine (Grant A3‐0317‐24‐429‐006).

## Conflicts of Interest

The authors declare no conflicts of interest.

## Supporting Information

Additional supporting information can be found online in the Supporting Information section.

## Supporting information


**Supporting Information 1** Table S1 Baseline clinical characteristics of included patients. Male, M. Female, F. Lumbar spinal stenosis, LSS. Procollagen type I N‐terminal propeptide, P1NP. C‐terminal telopeptide of type I collagen, β‐CTX. N‐MID Osteocalcin, N‐MID OC. 25‐Hydroxyvitamin D, 25(OH)D.

**Supporting Information 2** Form 1 | PT Purchase Application Form.

**Supporting Information 3** Figure S1 Ultra Performance Liquid Chromatography (UPLC) analysis of *Plastrum Testudinis* water extract. There were 26 active ingredients identified in the PT water extract using UPLC.

**Supporting Information 4** Table S2| Quantitative real‐time PCR primers.

**Supporting Information 5** Figure S2 Micro‐CT analysis of bone structure in the mouse tibial defect region. Supplementary 6A, Three‐dimensional micro‐CT reconstruction images of the tibial defect region. B, Pseudocolor images depicting bone tissue microstructure. C, Quantitative micro‐CT analysis of tibial defects in mice: bone volume/total volume (BV/TV, E), trabecular number (Tb.N), trabecular thickness (Tb.Th), and trabecular separation (Tb.Sp) (*n* = 6). Statistical significance was determined by one‐way ANOVA followed by Tukey’s post hoc test.  ^∗^ *p* < 0.05,  ^∗∗^ *p* < 0.01,  ^∗∗∗^ *p* < 0.001 vs. OTD group.

**Supporting Information 6** Figure S3 Osteogenic and osteoclastic activity in the mouse tibial bone defect region. Representative H&E staining images of the tibial defect region. Scale bars, 100 µm. (B) Representative TRAP staining images of the tibial defect region. Arrows indicate osteoclasts. Scale bars, 100 µm. (C) Representative immunohistochemical images showing RUNX2 expression in the tibial defect region. Scale bars, 200 µm. (D) Quantification of TRAP‐positive cells in each group (*n* = 6). (E) Quantitative analysis of RUNX2 immunohistochemical mean intensity in each group (*n* = 6).Statistical significance was determined by one‐way ANOVA followed by Tukey’s post hoc test or unpaired two‐tailed Student’s *t*‐test.  ^∗^ *p* < 0.05,  ^∗∗^ *p* < 0.01,  ^∗∗∗^ *p* < 0.001 vs OTD. ^###^ *p* < 0.001 for AMD group vs. P + A group.

## Data Availability

The data that support the findings of this study are available from the corresponding author upon reasonable request.
