# Supplementary material for: Plastrum Testudinis Extract Promotes Endogenous Bone Marrow Mesenchymal Stem Cell Migration in Osteoporotic Fracture Repair Partly by Activating the SDF‐1/CXCR4 Axis
Source: Stem Cells Int. 2026 May 19;2026:3033093. doi: 10.1155/sci/3033093 (PMC13184713; doi:10.1155/sci/3033093)
Supplement: Supplementary file 1 — Supporting Information 1 Table S1 Baseline clinical characteristics of included patients. Male, M. Female, F. Lumbar spinal stenosis, LSS. Procollagen type I N‐terminal propeptide, P1NP. C‐terminal telopeptide of type I collagen, β‐CTX. N‐MID Osteocalcin, N‐MID OC. 25‐Hydroxyvitamin D, 25(OH)D. [file SCI-2026-3033093-s001.docx]

**Supplementary** Table 1| Baseline clinical characteristics of included patients.

| ID | M/F | Age (y) | Menopausal Age (y) | Diagnosis | Creatinine (µmol/L) | Cystatin C (mg/L) | P1NP (ng/mL) | β-CTX(ng/mL) | N-MID OC (ng/mL) | 25(OH)D(ng/mL) | BMD of L1-L4 | BMD of Left Femoral Neck | BMD of Left Total Hip | T-score of L1-L4 | T-Left Femoral Neck | T-Left Total Hip | Z- L1-L4 | Z-Left Femoral Neck | Z-Left Total Hip |
| --- | --- | --- | --- | --- | --- | --- | --- | --- | --- | --- | --- | --- | --- | --- | --- | --- | --- | --- | --- |
| 1 | F | 68 | 53 | LSS | 55 | 0.89 | 39.43 | 0.479 | 11.8 | 27.31 | 0.824 | 0.681 | 0.753 | -3 | -2.6 | -2 | 0.4 | 0 | -0.1 |
| 2 | F | 72 | 52 | LSS | 72 | 1.17 | 75 | 0.382 | 18.88 | 30.72 | 0.604 | 0.492 | 0.553 | -4.7 | -3.9 | -3.6 | -0.4 | -0.5 | -0.6 |
| 3 | F | 69 | 52 | LSS | 64 | 0.97 | 129.9 | 1.25 | 25.69 | 22.54 | 0.893 | 0.633 | 0.743 | -2.4 | -2.9 | -2.1 | 0.4 | -0.8 | -0.5 |
| 4 | F | 64 | 53 | LSS | 90 | 0.87 | 33.36 | 0.25 | 12.4 | 12.93 | 0.952 | 0.825 | 0.937 | -1.9 | -1.5 | -0.6 | 0 | 0.3 | 0.5 |
| 5 | F | 57 | 51 | LSS | 114 | 1.13 | 53.07 | 0.472 | 15.53 | 40.74 | 0.964 | 0.898 | 0.916 | -1.8 | -1 | -0.7 | 0 | 0.9 | 0.4 |
| 6 | F | 70 | 52 | LSS | 92 | 0.97 | 63.36 | 0.497 | 12.94 | 65.94 | 1.18 | 0.789 | 0.843 | 0 | -1.8 | -1.3 | 1.6 | -0.3 | -0.7 |

Male, M. Female, F. Lumbar spinal stenosis, LSS. Procollagen type I N-terminal propeptide, P1NP. C-terminal telopeptide of type I collagen, β- CTX. N-MID Osteocalcin, N-MID OC. 25-Hydroxyvitamin D, 25(OH)D.
