# Supplementary figures and images for: Plastrum Testudinis Extract Promotes Endogenous Bone Marrow Mesenchymal Stem Cell Migration in Osteoporotic Fracture Repair Partly by Activating the SDF‐1/CXCR4 Axis
Source: Stem Cells Int. 2026 May 19;2026:3033093. doi: 10.1155/sci/3033093 (PMC13184713; doi:10.1155/sci/3033093)

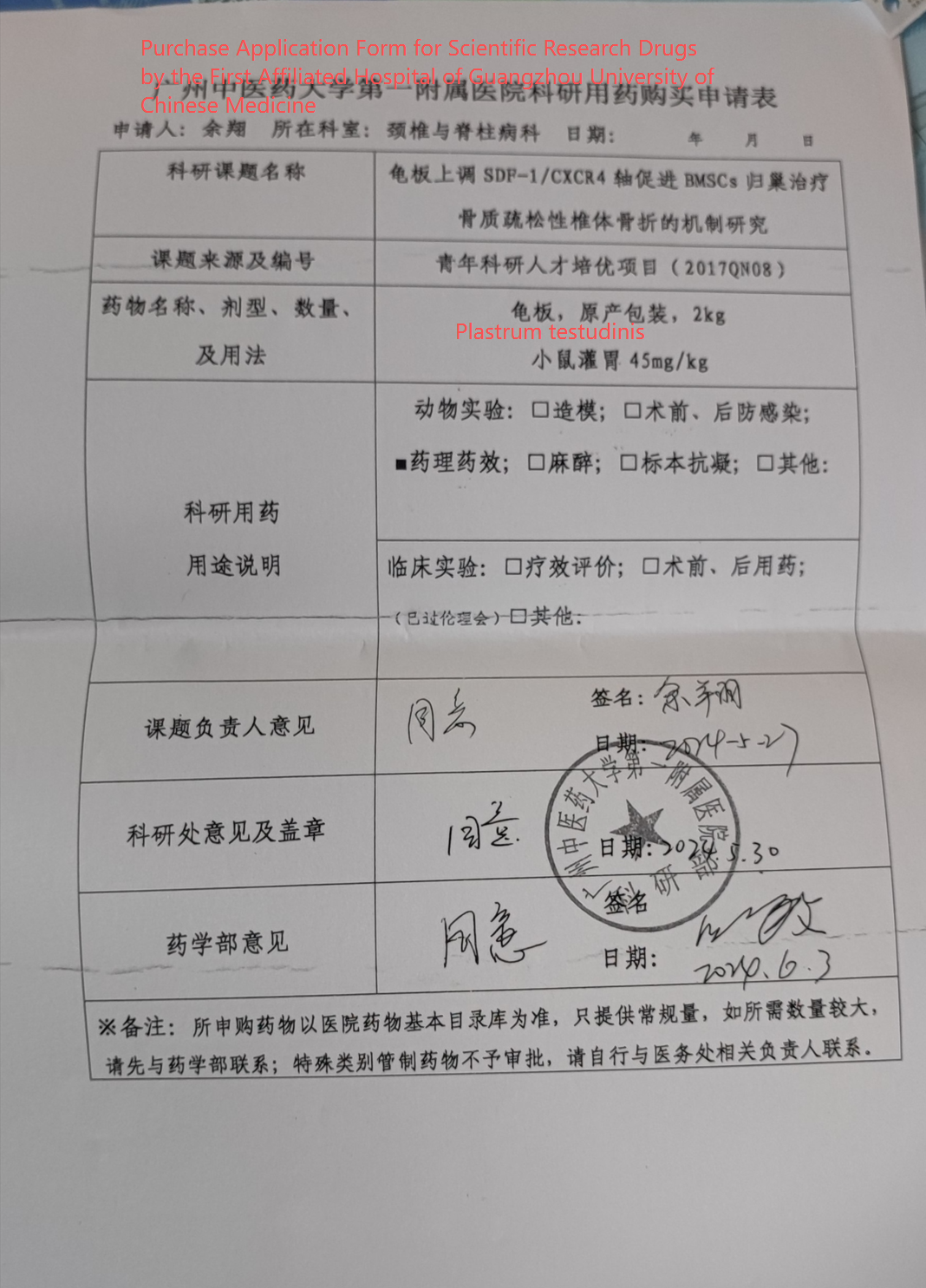


**Supplementary Form_1 | PT Purchase Application Form**

Supplement: Supplementary file 2 — Supporting Information 2 Form 1 | PT Purchase Application Form. [file SCI-2026-3033093-s002.docx]

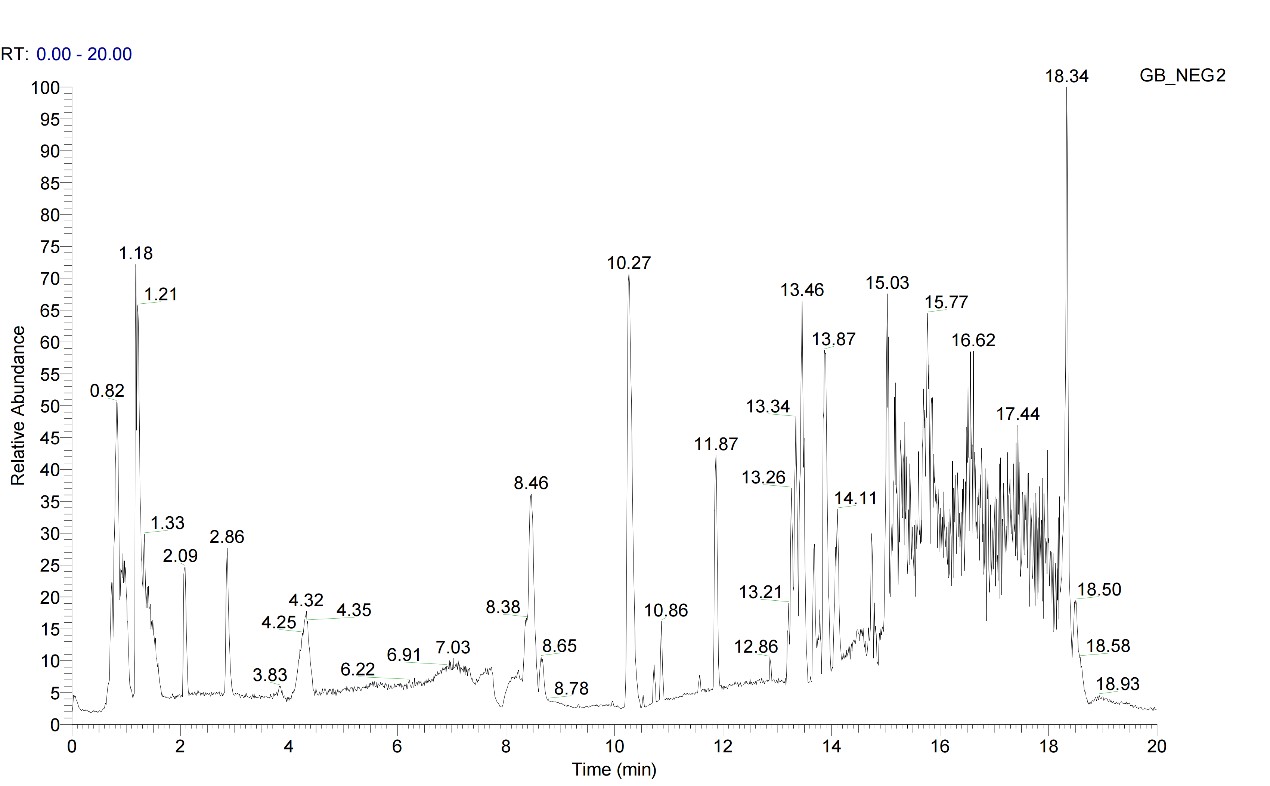

Supplement: Supplementary file 3 — Supporting Information 3 Figure S1 Ultra Performance Liquid Chromatography (UPLC) analysis of Plastrum Testudinis water extract. There were 26 active ingredients identified in the PT water extract using UPLC. [file SCI-2026-3033093-s003.jpg]

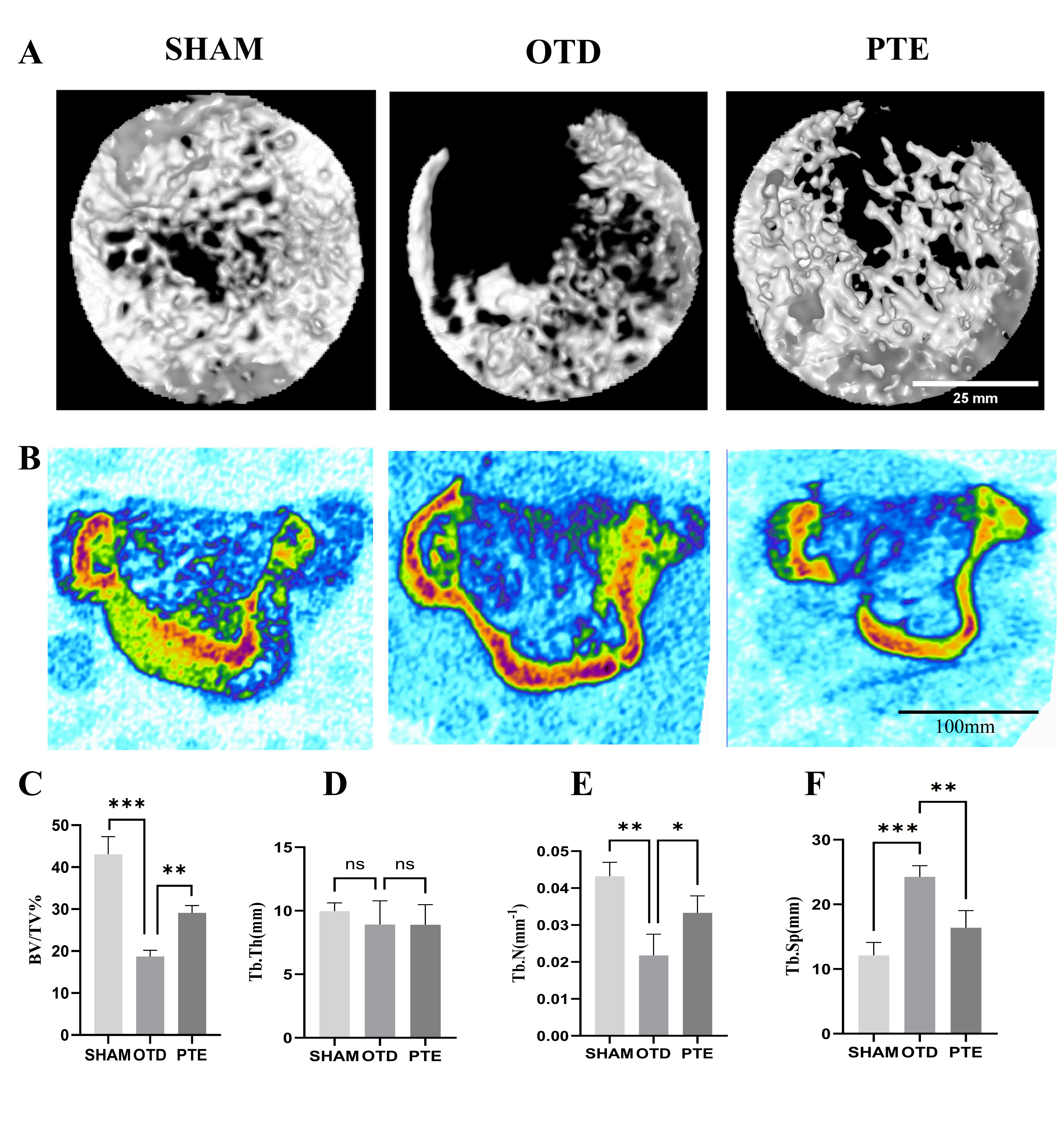

Supplement: Supplementary file 5 — Supporting Information 5 Figure S2 Micro‐CT analysis of bone structure in the mouse tibial defect region. Supplementary 6A, Three‐dimensional micro‐CT reconstruction images of the tibial defect region. B, Pseudocolor images depicting bone tissue microstructure. C, Quantitative micro‐CT analysis of tibial defects in mice: bone volume/total volume (BV/TV, E), trabecular number (Tb.N), trabecular thickness (Tb.Th), and trabecular separation (Tb.Sp) (n = 6). Statistical significance was determined by one‐way ANOVA followed by Tukey’s post hoc test. ∗ p < 0.05, ∗∗ p < 0.01, ∗∗∗ p < 0.001 vs. OTD group. [file SCI-2026-3033093-s005.jpg]

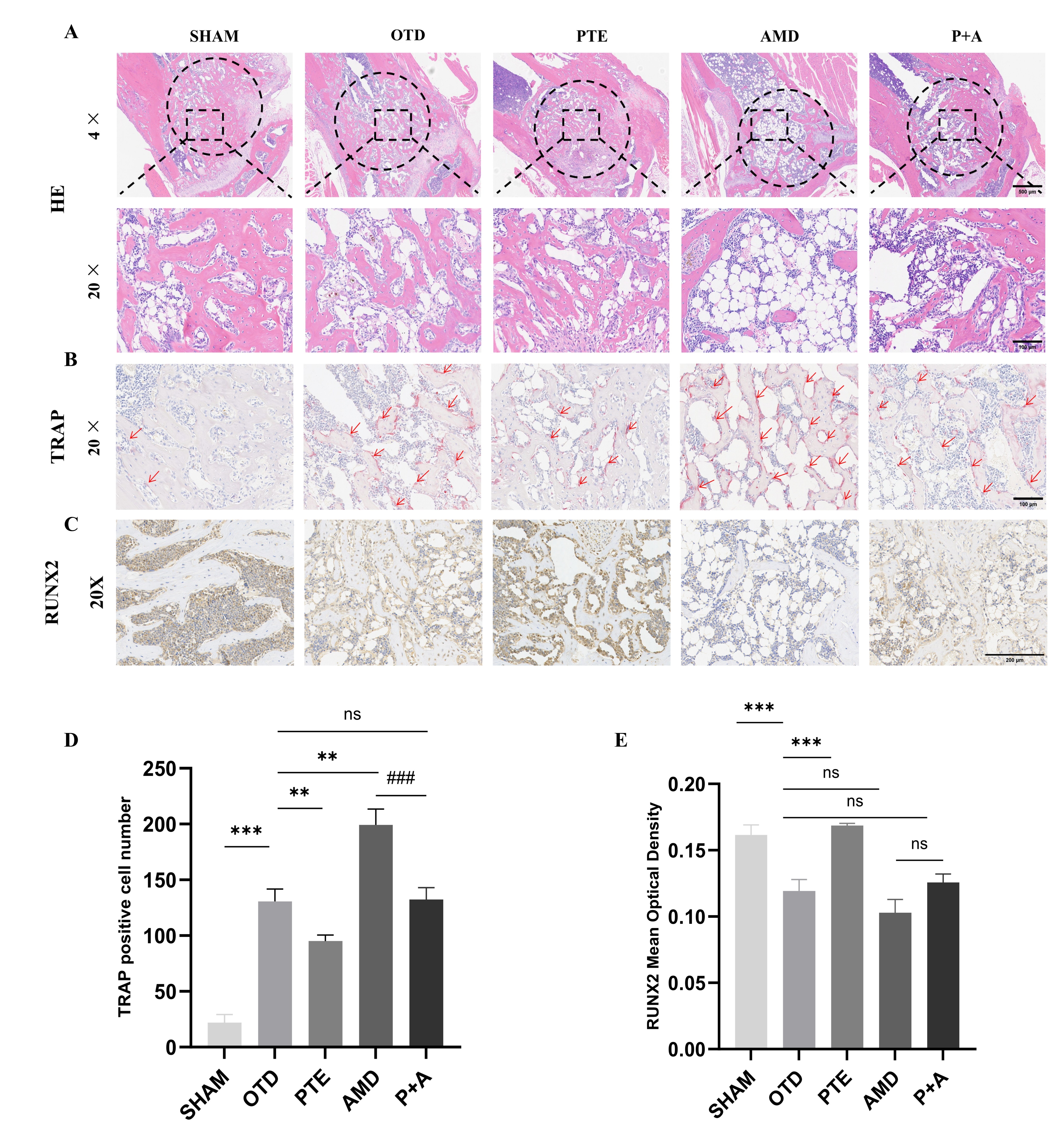

Supplement: Supplementary file 6 — Supporting Information 6 Figure S3 Osteogenic and osteoclastic activity in the mouse tibial bone defect region. Representative H&E staining images of the tibial defect region. Scale bars, 100 µm. (B) Representative TRAP staining images of the tibial defect region. Arrows indicate osteoclasts. Scale bars, 100 µm. (C) Representative immunohistochemical images showing RUNX2 expression in the tibial defect region. Scale bars, 200 µm. (D) Quantification of TRAP‐positive cells in each group (n = 6). (E) Quantitative analysis of RUNX2 immunohistochemical mean intensity in each group (n = 6).Statistical significance was determined by one‐way ANOVA followed by Tukey’s post hoc test or unpaired two‐tailed Student’s t‐test. ∗ p < 0.05, ∗∗ p < 0.01, ∗∗∗ p < 0.001 vs OTD. ### p < 0.001 for AMD group vs. P + A group. [file SCI-2026-3033093-s006.jpg]
