# Supplementary material for: Plastrum Testudinis Extract Promotes Endogenous Bone Marrow Mesenchymal Stem Cell Migration in Osteoporotic Fracture Repair Partly by Activating the SDF‐1/CXCR4 Axis
Source: Stem Cells Int. 2026 May 19;2026:3033093. doi: 10.1155/sci/3033093 (PMC13184713; doi:10.1155/sci/3033093)
Supplement: Supplementary file 4 — Supporting Information 4 Table S2| Quantitative real‐time PCR primers. [file SCI-2026-3033093-s004.docx]

**Supplementary** Table 2| Quantitative real-time PCR primers.

| Target genes | Primer Forward | Primer Reverse |
| --- | --- | --- |
| βactin | CCTCTATGACAACACAGT | AGCCACCAATCCACACAG |
| CXCR4 | AGTGACCCTCTGAGGCGTTTG | GAAGCAGGGTTCCTTGTTGGAGT |
| SDF-1 | TCGGTGTCCTCTTGCTGT C | GATGCTTGACGTTGGCTCTG |
